# Bibliographical

**Published:** 1898-11

**Authors:** 


### EDITORIAL.

### Bibliographical.

A TEXT-BOOK OF DENTAL PATHOLOGY AND THERAPEUTICS, INCLUDING PHARMACOLOGY. A Treatise on the Principles and Practice of Dental Medicine, for Students and Practitioners. By Henry H. Burchard, M.D., D.D.S.

This work consists of six sections and twenty-eight chapters, to which is added Section 7, Dental Pharmacology and Dental Materia Medica.

In Section 1 attention is given to Dental Pathology, its general principles, causes of disease, general and local; Bacteriology, with special reference to Dental Pathology and Therapeutics, together with infective inflammation, suppuration, etc., etc.

Section 2 presents Anatomy and the Development of the Teeth.

Section 3, Affections of Enamel and Dentin.

Section 4, Diseases of the Dental Pulp.

Section 5, Diseases of the Pericementum.

Section 6, Diseases of the Deciduous Teeth.

The arrangement of these subjects into comparatively brief chapters, for the most part at least, is a very good one indeed. The consideration of every subject presented is clear and understandable, even by the student, and is presented in an attractive manner.

Many subjects are brought out, and though briefly, yet clearly presented so that the reader is led on from subject to subject with pleasure, and with a desire to know what is next said.

Pathology, as it is sometimes presented and discussed, is not an attractive subject for study. In this work Dr. Burchard has succeeded in presenting his matter in such a manner as to remedy this difficulty. Dental Pathology and its allied subjects are here presented in such a way as to be helpful to every one, not only the student, but his teacher as well.

Dr. Burchard has been teaching and writing upon this subject for a number of years, and has given special attention to it and is doubtless as well equipped for the preparation of such work as any member of the profession, of this country. It is eminently worthy of a place as a text-book for the dental student and in the library of the practitioner; indeed, the physician will find much in it to interest and help him.

---

Oral Pathology and Practice. A text-book for the use of students in dental colleges and a hand-book for dental practitioners. By W. C. Barrett, M.D., D.D.S., M.D.S., Professor of Oral Pathology in the University of Buffalo, Medical Department; Professor of Dental Anatomy and Pathology in the Chicago College of Dental Surgery. Published by The S. S. White Dental Manufacturing Co., Philadelphia.

This work, just issued, is a very valuable one not because there has not been much written on the subject of Dental Pathology, but it presents the subject in its varied aspects in a very terse and understandable manner.

The Doctor in writing it says his object has been to condense not to amplify, that it may be published at as low a price as possible. With this in view cuts have been excluded. The work has thus been kept within the limits of a manual. It has been the aim of the author to consider as succintly as is consistent with clearness the fuctional derangements of all the oral tissues that properly fall within the compass of a broad dental practice. This work by Dr. Barrett is the outcome up to the present time of the experience and opinions formed in regard to the various subjects treated by him during the many years he was engaged in teaching these and kindred subjects.

The Doctor has been a thorough student and successful teacher, and is so familiar with the subjects embraced in this treatise that he has been able in a very thorough manner not only to present the essential points of the subjects treated, but to sift and leave out matter irrelevant and not necessary for a clear understanding of the subject.

It is oftentimes the case that works claiming to be written for text-books are overloaded with irrelevant matter, which is injurious rather than helpful to the student. This work is certainly to be commended for its terseness. Another feature of the work is the entire absence of cuts and illustrations.

We are pleased that the Doctor was bold enough to take this step. In some of our text-books illustrations have occupied a large percent of the space and many of them wholly unnecessary. It is a question of considerable importance whether this matter of illustrations has not been quite overdone and whether the student does not rely upon his impressions formed from the cuts, and to a goodly degree take the place of that which ought to be attained by intellectual effort, reasoning and understanding from language rather than illustrations. It is impossible to convey by cuts or illustrations anything like a full knowledge of any subject, and it is a question whether the impression made by the written or spoken language is not far more permanent and effective than can be made by any illustration.

Dr. Barrett has here set an example which ought to challenge the attention of both the authors and publishers. In some branches illustrations are admissable, and even advisable. There is, of course, a diversity of opinion on some of the subjects considered in this work. They are not, however, of such character as to call for any special criticism. There is very little that will lead the student astray. The personality of the author is quite apparent throughout the work. This, however, cannot be regarded as detracting from its merits.

This book should be adopted in all of our colleges as a text-book, and should also be found in the library of every dentist who cares to possess and read books pertaining to his chosen profession.

